# Examining the optimal factors that promote implementation and sustainability of a network intervention to alleviate loneliness in community contexts

**DOI:** 10.1111/hsc.13808

**Published:** 2022-04-26

**Authors:** Jaimie Ellis, Karina Kinsella, Elizabeth James, Tara Cheetham‐Blake, Madeleine Lambrou, Alexandra Ciccognani, Anne Rogers, Rebecca Band

**Affiliations:** ^1^ 7423 School of Health Sciences University of Southampton Southampton UK

**Keywords:** community, context, implementation, loneliness, network intervention, social isolation, sustainability

## Abstract

Community environments have the potential to alleviate loneliness and social isolation as they offer opportunity for sociality and to expand personal social network connections. Implementing a social network intervention in community environments to connect people to who are at risk of loneliness or social isolation could help alleviate these concerns. However, implementing interventions in community environments is made difficult by the interplay between the community context and intervention. Thus, to support implementation a detailed understanding of the types of community contexts is required. To examine the optimal factors that promote the implementation of a social network designed to alleviate loneliness and social isolation intervention in the community observations, interviews and documentary analysis were conducted. The Consolidated Framework for Implementation Research and a typology of community contexts were used to inform the data analysis and interpret the findings. Key factors were found to affect the implementation of the intervention in the different community contexts. These inter‐related factors operated across three domains. Service User Needs affected intervention take up as its suitability varied. The stability of the workforce and nature of everyday work also impacted on implementation. Finally, the fluctuating capacity of organisations and the organisational culture were also influential. No single community environment was found to have all of the optimal factors required for implementation and sustainably. The UK policy agenda of austerity had negatively affected community environments’ capacity to deliver such intervention through increasing service user needs and reducing available resources.

Trial registration: ISRCTN19193075.

**What is known about this topic?**

- Loneliness and social isolation are public health concerns in the UK and have been shown to be harmful to health and wellbeing.
- The role of community environments in alleviating loneliness and social isolation has been demonstrated.
- Context, the unique characteristics and circumstances, is influential to any implementation efforts, and literature calls for greater understanding of the role of context in implementation.

**What this paper adds?**

- A continuum of community environments with different characteristics and circumstances exists that affect implementation.
- No one type of community environment had all the necessary factors to achieve implementation and ultimately sustainability.
- The sustainability of an intervention to alleviate loneliness will be enhanced if community environments had greater human and financial resources.

## 1 INTRODUCTION

Alleviating loneliness and social isolation has become a priority in the UK where 45% of adults report feeling lonely occasionally, sometimes or often (ONS, 2020), and it is predicted that by 2025 up to two million people aged over 50 will experience loneliness (Age UK, 2020). Loneliness has a negative effect on physical and mental health (Coyle & Dugan, 2012; Leigh‐Hunt et al., 2017). Community environments have potential to alleviate loneliness and social isolation (Marmot et al., 2020). These environments provide opportunities for supported sociality (Mann et al., 2017), building new connections (Kennedy et al., 2016a) and community capital (Marmot et al., 2020), and have an important role to play in tackling loneliness and social isolation. This paper contributes towards the literature by examining how a social network intervention designed to alleviate loneliness and social isolation can be implemented community environments.

### 1.1 Implementation in community settings

The process of moving from evidencing an intervention is effective to implementing the intervention in practice has been described as a ‘leaky pipeline’ (Green et al., 2009). The research‐to‐practice gap can be minimised by gathering evidence in the context in which the intervention is destined (Green et al., 2009; Greenhalgh et al., 2017a). Context is the unique characteristics surrounding implementation efforts (Pfadenhauer et al., 2017) from the social, cultural, economic, political and legal and physical circumstances to the organisational circumstances (Peters et al., 2014). Context interacts with the intervention, and interventions have the potential to be shaped and transformed by the environment into which they are introduced (Hawe, 2015).

Evaluations of the implementation of complex interventions illuminate the need to understand interaction of the intervention, implementation site and the wider context (Armstrong et al., 2016; Greenhalgh et al., 2017a; Kennedy et al., 2016b). This is becomes especially pertinent when interventions are destined for community settings (Ellis, Vassilev, et al., 2020). Evaluations of implementations taking place in community contexts that draw attention to the importance of forming strong partnerships with stakeholders (Wurz et al., 2021). Using the Consolidated Framework for Implementation Research Brook and McGraw (Brook & McGraw, 2018) found that context was influential in the extent to which facilitators and participants engaged with the health coaching intervention, and their perception of the intervention. Payne et al.’s (2021) study found the familiarity community centres offered facilitated intervention uptake. Context then has the capacity to facilitate or inhibit the implementation of an intervention (Pfadenhauer et al., 2017), and thus it becomes pertinent to examine the contextual factors (Clarke et al., 2013; Damschroder et al., 2009; Greenhalgh & Papoutsi, 2018). This paper reports on the data gathered as part of an embedded process evaluation within a community‐based trial of a social network intervention to address loneliness and social isolation.

### 1.2 The trial

The study is a hybrid‐designed pragmatic randomised controlled trial (RCT). The hybrid‐design simultaneously assesses the effectiveness and cost‐effectiveness of a social network intervention (Genie intervention see Band et al., 2019). The social network intervention is a facilitated three‐staged tool. First, guided by conversation with a trained facilitator the user maps their personal community on a concentric circles diagram reflecting degrees of importance, placing those of most importance nearer the middle. Second, 13 questions are asked to identify the user's interest and needs. Thirdly, the intervention presents opportunities for social engagement by matching the user's identified interests and needs to local resources (i.e., services, groups and clubs). Using the user's postcode this information is presented on a map illustrating distance and details of the identified resource. The process evaluation sought to understand the wider socio‐political, organisational (community groups delivering the intervention) and individual (recipients of the intervention) factors that promoted integration and sustainability of the intervention. The focus of this paper is on the organisational level.

### 1.3 Aim

The aim of this study was to examine the factors that promote the implementation and sustainability of a social network intervention for loneliness and social isolation in community contexts.

## 2 DESIGN

This mixed‐methods study took an iterative approach to data collection, which provided the opportunity to study the implementation process, and for the unique aspects of each setting to be revealed.

Research questions:

1. Was the intervention implemented as planned?
2. How was the intervention incorporated into the way in which organisations reach, negotiate, work and sustain options for linking people to resources and connections?
3. How does the intervention impact on and become integrated into the organisation's capacity to provide options for people who are lonely/socially isolated?

Data collection was informed by the Consolidated Framework for Implementation Research. The five domains and 37 sub‐domains of the framework (Table 1) guided the pre‐implementation analysis (Ellis, Band, et al., 2020) and were carried forward to the implementation. Trial recruitment began October 2018 and was paused in March 2020 due to the COVID‐19 pandemic. The trial restarted on 1st October 2020 and is currently ongoing. This paper reports on the data gathered before March 2020.

**TABLE 1:** Consolidated framework for implementation research

CFIR domain	Sub‐concept
Intervention characteristics	Intervention Source Evidence strength and quality Relative advantage Adaptability Trialability Complexity Design quality and packaging Cost
Outer setting	Patient needs and resources Cosmopolitanism Peer Pressure External policies and incentives
Inner setting	Structural characteristics Networks and communications Culture Implementation climate (relative priority, organisational incentives and rewards, goals and feedback, learning climate) Readiness for implementation (leadership engagement, available resources, accessible information and knowledge)
Individual characteristics	Knowledge and beliefs about the intervention Self‐efficacy Individual stage of change Individual identification with organisation Other personal attributes
Process of implementation	Planning Engaging (opinion leaders, formally appointed internal implementation leaders, champions, external change agents) Executing Reflecting and evaluating

Abbreviation: CFIR, Consolidated Framework for Implementation Research.

### 2.1 Ethical considerations

The study was approved by the South‐Central Berkshire Health Research Authority and the University of Southampton's Research and Governance Ethics Committee.

### 2.2 Study setting

The study was delivered in collaboration with community partners in Southampton and Liverpool, UK. Partners were purposively contacted by the study team, or were referred in by a process of snowballing, if they had the potential to identify individuals at risk of social isolation or loneliness (Band et al., 2019). A total of 32 partners were recruited (Table 2). The partners have been categorised on a continuum across three typologies as developed in the pre‐implementation phase (Ellis, Band, et al., 2020). On one end of the continuum are partners who deliver services across a large geographical area and are known as Fully‐Professionalised Organisations (i.e., Statutory services). These partners supported individuals with a specific, identified need and would refer individuals to other services for additional or more prolonged support. In the middle are the organisations that tend to support individuals over a prolonged period and often in multiple ways (i.e., groups, support and information services). The Aspirational Community, Voluntary and Social Enterprise (hereon in known as ‘Aspirational Community partners’) operate at a regional to local level and are partially reliant upon voluntary income and volunteer involvement (i.e., local charities). At the other end of the continuum are Non‐Professionalised Community‐Based organisations (hereon in known as Community Based), which are typically faith‐based groups rooted in the local community and supported people in multiple ways, and the supported individuals were seen as being a member of the community. These groups heavily rely on volunteers and donations to function. Socio‐demographics of service users by typology are presented in Table 2.

**TABLE 2:** Typology continuum of community partners

Typology	Partner number	Partner description	Who facilitated			No. participants recruited^a^	Socio‐demographics		
			Them‐selves	[trial]	Other		Mean age	Live alone (yes)	Gender (female)
Fully professionalised organisations	1	Statutory service provider		/		77	52.34 (20.28)	21	57
	2	Statutory service provider	/			19	75.63 (9.92)	11	13
	3	Education provider		/		7	46.71 (13.56)	2	3
	4	Collection of GP practices		/		7	62.57 (19.68)	6	4
	5	Statutory service provider		/		5	71.00 (17.50)	4	1
	6	Private education provider		/		4	22.50 (4.04)	2	3
	7	Combined authority programme provider		/		11	44.18 (11.24)	1	10
	8	Housing Association	/			10	59.00 (9.32)	3	7
	9	International charity—local branch	/			3	86.33 (10.26)	3	2
	10	Charity		/		1	**	1	1
Aspirational Community, Voluntary and Social Enterprise	11	Group of charitable social enterprise		/		20	71.70 (11.84)	16	18
	12	Private education provider	/			2	41.00 (22.62)	1	1
	13	Healthy living centre		/		1	**	1	1
	14	Community interest company (CiC)	/			5	60.00 (13.50)	4	1
	15	National charity—local branch	/			10	70.10 (12.18)	9	7
	16	National charity—local branch		/		3	65.33 (4.04)	2	2
	17	Charity—city based		/		19	67.11 (13.54)	15	10
	18	Charity—city based	/			11	79.00 (12.49)	9	10
	19	Council for voluntary services	/	/		8	69.50 (14.15)	7	3
	20	Charity—city based				0	—	—	—
	21	Community recovery project		/		2	39.00 (1.41)	0	0
	22	Charity—city based		/		0	—	—	—
	23	Charity—city based	/	/		13	73.50 (12.37)	10	9
	24	Community centre—registered CiC	/			12	42.67 (10.76)	2	6
	25	Charity Food Bank		/		10	37.70 (7.74)	0	10
	26	Community association		/		10	68.00 (12.69)	4	6
	27	Community centre		/		3	63.33 (6.11)	3	1
	28	Community centre—charity registered	/			8	41.63 (14.74)	1	7
Community based	29	Church	/			29	67.69 (15.40)	10	20
	30	Charity—city based	/			7	71.43 (12.39)	4	7
	31	Mosque			/	1	**	0	1
	32	Church		/		0	—	—	—

Note

AGE is constant when Organisation recruited [**] has been omitted.

^a^ Total of all individuals consented into the study during reporting period; includes withdrawals.

**FIGURE 1:**
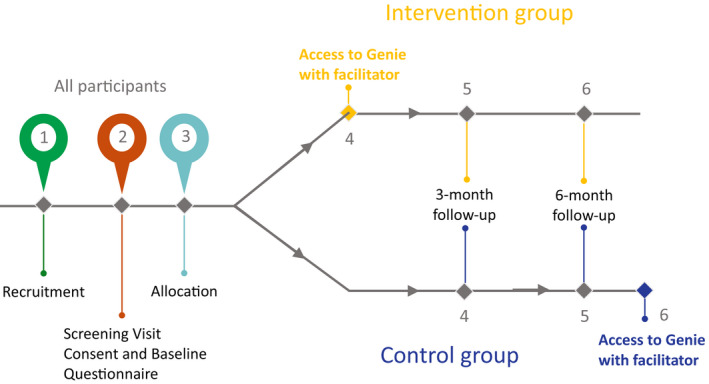
Work‐flow and division of labour

Each partner represents an implementation site and the study team worked with the partners to deliver the RCT. The proposed division of labour (Figure 1) suggested partners were to be responsible for identifying and allocating members of the workforce to become intervention facilitators, identifying and recruiting participants to receive the intervention, as well as delivering the intervention (immediately after baseline for intervention group and after 6 months for control group). Partners were also responsible for conducting a follow‐up social network mapping exercise at 3 months for the intervention group. The study team was responsible for each screen visit, obtaining consent, completing the baseline questionnaire and randomisation of participants. The specific implementation plans and how the tasks were fulfilled were decided by the partner. These differences are reported here.

### 2.3 Methods

Three methods were used to illuminate the organisational work practices and capacity of partners and the effect of implementation on these and the engagement with the intervention.

Observations occurred continually throughout the data collection period and were made by JE and supported by KK, AC, TCB and ML. To understand the partners’ contexts, the meetings between the study team and partners were observed. Site visits took place with all partners. Meeting notes were taken in situ and reflective notes immediately after.

All correspondence with partners was recorded and contributed towards the data collected. Notes from telephone conversations were recorded directly after the call. Other documents, such as leaflets and newsletters produced by the organisation, were also collected.

All organisations were invited to interview via email/call, and in total 19 interviews were conducted across 12 partners, spanning the continuum, and included managers and employees/volunteers. The interview guide was developed with consideration of the Consolidate Framework for Implementation Research. However, the guide was used flexibly to explore points of interest as they arose. Interviews were carried out in person or by phone by JE, KK, AC or TCB, lasted between 30 and 40 min, were audio‐recorded and transcribed verbatim. Reflective notes were made after each interview to capture non‐verbal elements.

### 2.4 Analysis

Data analysis was informed by Layder's adaptive theory (Layder, 1998). A process of familiarisation and coding (inductively and deductively) was applied. Deductive coding drew on the Consolidate Framework for Implementation Research and the typology of community contexts (Ellis, Band, et al., 2020). Through a process of inductive coding, concepts from the framework were helpful in understanding the factors important to implementation and sustainability. In order to highlight the contextual sensitivities, the typology of community contexts was used to inform the analysis by linking the framework's concepts to each typology. In keeping with the approach taken, data analysis was an iterative process that helped to ground onward data collection. JE led the data analysis and all authors sense checked the analysis and interpretation of the data that is ported here.

## 3 FINDINGS

Several factors influenced the implementation and sustainability (over the project duration) of the intervention that were inter‐related and operated across three domains; Service User, Workforce and Organisational Structure. To draw out the elements and position them with community contexts, the findings are structured according to these three domains. The letters I, O, D are used to indicate the data source (Interviews, Observations and Documents respectively). The letter P followed by a number to indicate a community partner as displayed in Table 2.

### 3.1 Service users

To address the factors relevant to implementing an intervention to alleviate loneliness and social isolation, it is important to understand the nature of loneliness and social isolation. Specifically, understanding the needs of those people experiencing, or who are at risk of experiencing, loneliness and social isolation. The term ‘service users’ refers to the individuals who were accessing support from the community partners. The concept ‘patient needs’ (Damschroder et al., 2009) is relevant here as the needs of the service user and their ability to engage with the intervention speaks to the reach of the community partners.

The needs of the service user had a baring on whether the intervention designed to alleviate loneliness and social isolation was suitable. This point is relevant to all typologies. However, the example of Aspirational Community partners illustrates that service user needs were complex, i.e., there were multiple needs such as housing, financial and health needs, the intervention was deemed unsuitable.

… when people are firefighting, when they are sorting out housing, food, money, it’s hard to think about the bigger stuff like isolation. It [intervention] is better when people are more stable. (O:P17)

Whilst Aspirational Community partners, like all the typologies, were in contact with people experiencing loneliness and social isolation, service users being supported by Aspirational Community partners often had more urgent needs that precluded them from being able to participate.

People may not be engaging because they are coping with housing issues, debt, drugs and alcohol, children not attending school. (O:P14)

The service user's needs that prevented them from engaging might also be considered as contributing factors towards loneliness and social isolation, however, when people have multiple needs ‘loneliness was a tiny part of the bigger picture’ (I:P10), and thus the intervention designed to help with loneliness and social isolation was not a priority. Where service users from Aspirational Community partners did want to engage, the success of the intervention was affected by the user's socioeconomic circumstances, which could prevent them from fully benefiting from what was offered.

some of the young people find it hard to communicate and socialise with others, money is a really big barrier so people don’t necessarily have the money to go and do what they want to do. (O:P14)

Due to the needs of the service users accessing the services of Aspirational Community partners, the reach of this typology was restricted as the intervention was unsuitable, in part or wholly, for the users. The rising demand for support and increasing complexity of service user needs had a second effect on implementation for the Aspirational Community partners. These factors placed strain on the partners’ capacity to deliver the intervention. Specifically, the strain on capacity meant the threshold to access support increased, and Aspirational Community partners were having to prioritise their resources to those in most need.

People used to call up because they were a little lonely and they used to send a befriender [but] now they can't do that, people have to be really lonely. (O:P17)

The strain on capacity meant that although Aspirational Community partners could identify individuals who were experiencing loneliness/social isolation, they were only able to support the most severe cases. The implementation across all typologies was affected by the wider socio‐political context and the effect of this was seen most clearly in the Aspirational Community partners who saw increased demand from service users.

Especially [difficult] since the council and social care is changing and there is no preventative work going on. (O:P17)

The rising needs of service users were also found to affect implementation in some of the Fully‐Professionalised partners’ contexts.

[P5] are a community facing service whose referrals come via the GP. [facilitator] explained that although they had tried to engage their population with it [intervention], they felt their population was too much in crisis to take part. (O:P5)

Where the intervention and service user need could not be reconciled, i.e., Partner 5, this became a contributing factor towards the partner withdrawing from the study.

It is important to understand service user needs in each community typology. The most Fully‐Professionalised partners offer a contrasting experience to what has been outlined above. That is, the reach of the most Fully‐Professionalised partners were supported in part due to the intervention being an addition to existing services, and as such it was also considered to align more appropriately to the service users’ needs.

Actually, I guess there was a need, it felt like people wanted more support than just the service [they] wanted to connect and perhaps were feeling isolated. (I:P1)

In examples like this, the service users accessing the Fully‐Professional partners were willing and crucially, unlike the more Aspirational Community partners, were more able to engage with the intervention. This meant the very most Fully‐Professionalised partners had exceptional reach compared with the Aspirational Community partners.

Finally, the more Community‐Based partners were found to have service users with a range of needs, which meant the intervention was suitable for some and not others. However, these partners also had reduced reach as they tended to support a small geographical location that limited the number of people accessing the organisation.

The needs of the service users, and particularly understanding how loneliness and social isolation are experienced were the key factors affecting implementation for they determined the suitability of the intervention. However, the service users sat within a larger system; the partner's broader organisational system, which also impacted implementation.

### 3.2 Workforce

Working with services users was a range of personnel, both paid and unpaid, and the term ‘workforce’ refers to these individuals. ‘Workforce’ differs slightly from the domain ‘characteristics of the individual’ in that it is more than any one individual but rather refers to what is required at the level of the collective workforce. The nature of everyday work, (re)socialisation of the workforce and workforce depth were the factors affecting the implementation of the intervention.

Delivery of any intervention requires training to upskill the workforce; however, training and upskilling the workforce specifically on how to support people with loneliness and social isolation was important to implementation success. Particularly so because a significant part of the intervention was relational and required good inter‐personal skills on behalf of the facilitator. The first stage of the intervention where the facilitator guides the user to map and talk through their personal network lends itself more favourably to the roles and individuals with experience of relational working. Implementation was more successful where this experience of working closely with service users aligned with the nature of the service's everyday work. The reverse of this was seen in Fully‐Professionalised partners who were able to recruit to the study but not facilitate the intervention. The everyday work of these partners was more referral based, which negatively affected implementation because the style of working with service users was different.

In contrast the Aspirational Community and Community‐Based partners’ everyday work was relational, which aligned with the intervention and supported implementation.

My role as a link worker made the project a very natural fit into my existing role. (D:P19)

Where the intervention work aligned more closely with the everyday work of the community partner implementation was easier to progress, and the alignment arguably also strengthened sustainability.

We did it because it is what we do anyway, but we have the ability to go with people. (O:P18)

Having a natural alignment between intervention and everyday work promoted implementation by giving the community partners’ workforce the experience necessary to be able to facilitate the intervention. Where this was not the case, a process of (re)socialisation was required. (Re)socialisation includes, but is not limited to, intervention‐specific training but also changes to work practices. As in the example of the Fully‐Professionalised partners who were more bureaucratic and rigid in work practices, they required (re)socialising into more dynamic work practices to promote implementation.

some of our research staff are very confident in delivering research but there is a strong sense of things being right and wrong. And I think that the modelling process helps the data collectors and, you know, whoever it is to carry the approach of the study team and it helps to bring to life the research document. (I:P1)

(Re)socialisation was more relevant for the more Fully‐Professionalised partners because there was less alignment between the intervention and the everyday work. (Re)socialisation was required to find flexibility in the context and adjust established ways of working, which when coupled with flexibility with the study team helped to embed the intervention work. For example, five Fully‐Professionalised partners successfully recruited individuals to the study but were unable to facilitate the intervention (Table 2).

In finding that flexibility in work practices and relational working were factors influencing implementation, this sheds light on who is best placed to facilitate an intervention to alleviate loneliness and social isolation. For the Aspirational Community partners, however, their reliance on volunteers and their precarious nature of their workforce hampered implementation. What was particularly unique to the Aspirational Community and Community‐Based partners was their reliance on volunteers. This impacted implementation in two ways. The first related to skills required to deliver the intervention as volunteers experienced low confidence in their capabilities to facilitate.

our volunteers will worry about whether or not they could actually (a) do it and then (b) carry the commitment through. (I:P23)

The second relates to the precarious nature of relying on a volunteer workforce, and the speed at which this can diminish. This was seen in the example of a Community‐Based partner (Partner 29) who relied solely on one volunteer who moved away from the trial area, which resulted in a complete absence of workforce, and thus the intervention was unsustainable. Losing a member of the workforce for Aspirational Community partners stretched capacity of the partner and thus disrupted implementation.

I just needed more, almost, like, time, and that's where my colleague's role completely and utterly did a 360 and she moved away from the visiting scheme. (O:P18)

The workforce turnover was experienced by all partners, but where members left the Fully‐Professionalised partners they were more easily replaced.

as we were leaving the office we were introduced to [Male] who will replace [Female] as the facilitator. [Study] had already been written into his objectives for the next quarter. (O:P8)

The Fully‐Professionalised partners required (re)socialisation to achieve implementation, whereas the everyday work of Aspirational Community partners had more natural alignment with the intervention. Implementation efforts were affected by workforce depth, but the Fully‐Professionalised contexts were more resilient at overcoming this.

### 3.3 Organisational structure

The term ‘organisational structure’ relates to the concepts ‘inner setting’ domain, and refers here to the two factors found to influence implementation: the culture and capacity of the organisation.

Community organisations have the potential to reach people experiencing loneliness and social isolation as outlined. However, a significant factor affecting implementation for all partners was the issue of capacity, which was found to fluctuate across the entire typology of community partners.

I think that it would have been at full capacity whether that was from me or from other people, I guess because of recruitment as well at the same time. Yeah, issue of capacity. (I:P1)

That being said, the issue of capacity most blighted the Aspirational‐Community and Community‐Based partners.

We integrated a whole load of services together, basically, and then we had … We had leaders for everything, but, unfortunately, one of the team leaders had to take emergency leave, and it was just all a bit crazy. And then we identified some gaps, and we filled those gaps, but then we didn’t have the money to fill other gaps, and, yeah. So, it was testing times, as always. (I:P18)

The capacity of these partners fluctuated due to a combination of workforce turnover, increasing service user needs and issues around funding. Funding cycles were found to affect the Aspirational Community partners because of their reliance on funding from charities, clinical commissioning groups and local/national governments. Often funding was tied to delivering a set of defined objectives over a period of time, which made implementing tasks outside of those contributing towards performance indicators and funding objectives difficult.

As [P19] develops, it is difficult for me to stay whether the organisation would or wouldn’t have capacity as this would depend on the various other projects that the team are working on. (D:P19)

The reliance on securing funding for the more Aspirational‐Community partners also meant they felt obliged to prioritise the (additional) demands of funders despite this stretching capacity further.

In January the CCG asked [P15] to go into A+E because people have been waiting so long and they go and make tea and drinks. They said they felt they ‘had to do this, because you do not say no to the commissioners, especially when the tendering process is up for renewal’. (O:P15)

The need to respond in an accountable way to funders meant the intervention work, which was not included in performance indicators, was not prioritised. The commissioning cycles and tender process affected implementation, often delaying progress as resources were diverted elsewhere, and they were also detrimental to sustainability as Aspirational Community partners were fighting for survival.

The current funding, from the housing association and Children in Need is running out. There are different funding pots with different time scales but no core funding to maintain sustainability. (O:P24)

The fluctuating capacity and precarious nature of contexts were significant factors affecting implementation and sustainability, as was culture.

As has been outlined, the everyday work of the Aspirational Community partners aligned with the intervention, and this was in part due to the culture of these organisations. Culture, the values and expectations of the organisation, supports implementation where alignment between the culture and the intervention exists, as seen with the Aspirational Community partners.

it really did fit very well with our ethos. It was mostly about how then do we make it come into being. (I:P23)

The Aspirational Community and Community‐Based partners were more likely to have been established in response to an identified need in the local community, and these founding values were reflected in the culture.

[location] was in the top 64 loneliest places in the UK, she saw this and saw her church was in the middle and thought ‘as a church we need to do better, we need to reach out and show these people love’. (O:P30)

Being value‐driven drove a commitment to pursuing implementation. Although these partners often had few resources and were often operating at capacity, their value‐driven culture was influential in sustaining the pursuit of implementation.

[Name] said that she feels like ‘they collude with statutory services because they know they will do it anyway … . Because the voluntary sector have a different value base … because we see the face standing in front of us and we won’t turn them away’. (O:P17)

The culture of the Fully‐Professionalised partners was more bureaucratic, which, as discussed, required some level of (re)socialisation in order to promote implementation.

Culture and capacity affected implementation in different ways and were significant for the Aspirational Community and Community‐Based partners. However, issues of capacity for these partners were often overcome by the culture that drove the commitment to an intervention designed to alleviate loneliness and social isolation.

## 4 DISCUSSION

This paper set out to explore the factors that promote implementation and sustainability of an intervention designed to alleviate loneliness and social isolation in community settings. Establishing how to implement and sustain interventions in community settings is key to addressing loneliness and social isolation because access to community resources is important to finding a way‐out loneliness. This is especially so in the UK context where the health policy focus emphasises the role of community and voluntary groups in health prevention and care (NHS England, 2019). What has been illustrated is that no single community typology possessed all of the factors required for implementation and sustainability.

The findings presented illuminate the need to be sensitive to the context; a point made in the implementation literature (Ellis, Vassilev, et al., 2020; Green et al., 2009; Greenhalgh et al., 2017a). This paper goes further in illustrating how community settings vary and understanding each typology is important. In the context of loneliness and social isolation what has been demonstrated is the Fully‐Professionalised organisations have the resources and stable workforce, factors that Milat et al. (2013) identify as important to adoption of interventions, but the everyday work of these organisations does not align in a way, which means these organisations can easily deliver an intervention designed to alleviate loneliness and social isolation. Whereas the Aspirational Community organisations were found to have greater alignment and experience of delivering health and well‐being support interventions, thus did not require (re)socialisation in order to be able to support people experiencing loneliness/social isolation. However, although these organisations had expertise in supporting people with loneliness/social isolation, they saw a rise in the number of people with complex needs. This impacted on the intervention suitability. This illuminates the importance of alignment between user‐need and the intervention for successful implementation and ultimately sustainability. Greenhalgh et al. (2017b) point to the importance of understanding the condition as an influential factor in the non‐adoption and abandonment of an intervention, and this study offers support to this.

The findings also shed light on the difficulty of implementation in a socio‐political context of an austerity agenda. The policy agenda contributes towards shaping the socioeconomic circumstances of both the individuals for whom the intervention is intended in the community settings. Austerity has led to an increase in demand for support from community organisations as service users’ needs grow in complexity. In the context of loneliness and social isolation, as has been demonstrated here, an intervention to address this has a declining suitability as service‐user needs increase. The negative impact of the austerity agenda on implementation has been reported (Ellis, Vassilev, et al., 2020). The experience here builds on this by illustrating how implementation in the context of austerity negatively affects the setting's capacity in numerous and interacting ways. Not least the precarious nature of many community settings, who alongside providing support to individuals, are continuously seeking financial investment to secure their own survival. The cuts in funding to community and voluntary settings have resulted in a widening of health inequalities (Marmot et al., 2020), and as illustrated here, the need for these organisations is increasing as their ability to respond is declining.

### 4.1 Implications and study limitations

This study is limited by its focus on an intervention for loneliness and social isolation within a UK community context. It is acknowledged that the role of community settings in health and well‐being support is likely to differ for readers outside of the UK. The implications however speak more generally to the need to understand each unique setting. This relates to Bunce et al.’s (2020) call to understand context‐specific pathways to implementation, and Greenhalgh et al.’s (2017a) call for studies to be locally situated. The typology of community settings, along with the Consolidated Framework for Implementation Research, was a useful tool for understanding the nuances of each setting. Community settings were different and implementation required a personalised approach. It is recommended that any implementation team would benefit from embedding themselves in the setting to understand the nuances and search for the flex. Furthermore, there are implications for the carrying out of RCTs. Specifically, RCTs can be both methodologically rigorous and locally situated, which can help to bridge the lag between evidence and implementation (Milat et al., 2013). Through the use of immersive, context sensitive research methods to aid understanding, the gathering of evidence and design of implementation strategy can be achieved simultaneously.

## 5 CONCLUSIONS

The concluding point returns to the issue of austerity. Firstly, this brings into question the matter of sustainability and the role of community settings in the delivery of health and social care. Sustainability of a public health intervention is likely to be hampered by a political context that is stripping the sector of available resources and financial support at time when service user's needs are increasing. The second point relates specifically to implementing an intervention designed to tackle loneliness and social isolation in such a political context, and the difficulties of this. It is especially important because these identified public health issues are just a few of the mounting needs of individuals. Implementing an intervention to tackle a public health issue requires not only a detailed understanding of the setting in which they are destined, but it also requires a joined‐up approach with socioeconomic, education, health and well‐being measures to ensure any public health intervention is suitable and relevant to user needs.

## CONFLICT OF INTEREST

The authors have no competing interests to declare.

## AUTHOR CONTRIBUTIONS

JE has led the process evaluation on which this paper is based. RB developed the initial idea for the PALS study and obtained funding in collaboration with AR. JE collected the process evaluation data with support from KK, AC, EJ, TCB and ML. JE led the data analysis and produced the initial paper draft, with all authors contributing towards subsequent drafts. All authors have approved the final version.

## ETHICAL APPROVAL AND CONSENT TO PARTICIPATE

Ethical approval for the PALS study has been obtained from the South Central‐Berkshire ethics committee (reference: 15/SC/0245). All substantial amendments must be approved by the University ethics committee and NHS ethics committee responsible for the trial, in addition to approval by HRA. Investigators are kept up to date with relevant changes via regular management group meetings. Written consent was gained from participants prior to data collection.

## CONSENT FOR PUBLICATION

Written consent was given by participants for data to be used in publications arising from this study.

## Data Availability

The datasets analysed during the current study are available from the corresponding author on reasonable request.
